# Comparative Clinical Outcomes of Robotic-Assisted and Conventional Total Knee Arthroplasty: A Retrospective Cohort Study

**DOI:** 10.7759/cureus.111993

**Published:** 2026-07-03

**Authors:** Ankit Kumar Garg, Anil Kumar Karpetee, Ravi Diwakar, Hishanil Rasheed, Kischentaran R Sanmugam, Priyanka Purohit, Sajidali S Saiyad

**Affiliations:** 1 Orthopaedics, ITSA Hospitals, Raipur, IND; 2 Orthopaedics, Netaji Subhash Chandra Bose (NSCB) Medical College, Jabalpur, IND; 3 Orthopaedic Surgery, Virendra Kumar Sakhlecha Government Medical College, Neemuch, IND; 4 Orthopaedics and Trauma, Yenepoya Medical Hospital, Mangalore, IND; 5 Trauma and Orthopaedics, NHS Tayside, Dundee, GBR; 6 Obstetrics and Gynaecology, Dr. N D Desai Faculty of Medical Sciences and Research, Nadiad, IND; 7 Physiology, Pacific Medical College and Hospital, Pacific Medical University, Udaipur, IND

**Keywords:** arthroplasty outcomes, conventional total knee arthroplasty, enhanced recovery after surgery, functional outcomes, knee osteoarthritis, patient-reported outcome measures, precision orthopedics, propensity score matching, robotic-assisted total knee arthroplasty, robotic surgery

## Abstract

Background

Robotic-assisted total knee arthroplasty (RA-TKA) has emerged as an innovative technology aimed at improving surgical precision and optimizing postoperative outcomes. However, whether these technical advantages translate into meaningful clinical benefits compared with conventional total knee arthroplasty (C-TKA) remains uncertain.

Objective

To compare early postoperative functional recovery (primary outcome) and perioperative outcomes, complications, revision surgery, and patient satisfaction (secondary outcomes) over 12 months in patients undergoing primary unilateral total knee arthroplasty (TKA) using robotic-assisted or conventional techniques.

Methods

This retrospective cohort study included 301 patients who underwent primary TKA at a specialized orthopedic center over a two-year period. Of these, 87 patients underwent RA-TKA and 214 underwent C-TKA. Baseline demographic and clinical characteristics were comparable between groups. Perioperative outcomes, patient-reported outcome measures, complications, and satisfaction rates were evaluated. Multivariable linear regression and propensity score matching (1:2 nearest-neighbor matching) were performed to identify independent predictors of recovery and reduce selection bias.

Results

Robotic-assisted TKA was associated with significantly longer operative time (108.4 ± 14.2 vs. 88.6 ± 11.5 minutes; p<0.001) but demonstrated lower blood loss (255 ± 92 vs. 289 ± 105 mL; p=0.03), reduced postoperative hemoglobin decline (1.9 ± 0.7 vs. 2.2 ± 0.8 g/dL; p=0.01), and shorter hospital stay (4.2 ± 1.1 vs. 4.8 ± 1.3 days; p=0.01). Statistically significant improvements in early functional outcomes were observed in the robotic cohort at 6 weeks and 3 months; however, none of these differences exceeded established minimal clinically important difference (MCID) thresholds. At 12 months, functional outcomes were comparable between groups. Complication rates, revision surgery, and patient satisfaction did not differ significantly. Robotic-assisted TKA remained an independent predictor of improved three-month Knee Society Score following multivariable adjustment and propensity score matching.

Conclusions

RA-TKA provides superior early postoperative recovery, reduced perioperative morbidity, and shorter hospitalization compared with conventional TKA. No statistically significant differences were observed in complications or one-year clinical outcomes, although the study was underpowered to detect small differences in rare adverse events.

## Introduction

Total knee arthroplasty (TKA) is widely recognized as one of the most effective surgical interventions for end-stage knee osteoarthritis, providing substantial pain relief, restoration of function, and improvement in quality of life. As the global prevalence of knee osteoarthritis continues to rise with increasing life expectancy and obesity rates, the demand for TKA is expected to grow substantially in the coming decades [1,2]. Despite the overall success of the procedure, approximately 15-20% of patients remain dissatisfied following surgery, often due to persistent pain, functional limitations, or unmet expectations [3]. Consequently, considerable efforts have been directed toward improving surgical precision and optimizing postoperative outcomes.

Conventional TKA relies on manual instrumentation and surgeon experience to achieve accurate component positioning, restoration of limb alignment, and soft-tissue balancing. Although this approach has demonstrated excellent long-term survivorship, variability in implant placement and alignment remains a recognized challenge. Suboptimal component positioning has been associated with altered biomechanics, increased polyethylene wear, instability, and reduced implant longevity [2,4]. In response to these limitations, robotic-assisted total knee arthroplasty (RA-TKA) has emerged as an innovative technology designed to improve surgical accuracy through three-dimensional preoperative planning, intraoperative guidance, and real-time assessment of implant positioning and soft-tissue balance [4,5].

Recent evidence suggests that robotic-assisted systems can achieve more precise implant alignment and reduce the frequency of mechanical-axis outliers compared with conventional techniques [5-7]. Systematic reviews and meta-analyses have consistently demonstrated superior radiographic accuracy with RA-TKA, supporting its potential to enhance surgical reproducibility and reduce technical variability [5,6]. Furthermore, robotic platforms facilitate individualized alignment strategies and dynamic gap balancing, enabling a more patient-specific approach to knee reconstruction [2,7].

However, whether these technical advantages translate into meaningful clinical benefits remains uncertain. Although improved alignment accuracy has been consistently reported, several contemporary meta-analyses have found limited or inconsistent differences between robotic-assisted and conventional TKA with respect to patient-reported outcome measures, range of motion, complication rates, and short- to medium-term functional recovery [5-8]. In addition, robotic procedures are often associated with longer operative times, increased infrastructure costs, and a learning curve that may influence perioperative efficiency [1,6]. Real-world studies have also highlighted the need for further evidence evaluating the effectiveness of robotic systems in routine clinical practice outside highly specialized centers [7].

Given these ongoing uncertainties, additional comparative investigations are needed to determine the clinical value of robotic assistance in TKA. Therefore, this retrospective cohort study aimed to compare early postoperative functional recovery at six weeks and three months as the primary outcome between robotic-assisted and conventional total knee arthroplasty (C-TKA) in patients undergoing primary unilateral TKA. Secondary outcomes included perioperative parameters, one-year functional outcomes, complications, revision surgery, length of hospital stay, and patient satisfaction over a minimum follow-up of 12 months.

## Materials and methods

Study design and setting

This retrospective cohort study was conducted at a dedicated orthopedic specialty hospital in Udaipur, Rajasthan, India, to compare clinical outcomes following RA-TKA and C-TKA. The study evaluated patients who underwent primary TKA over a two-year period preceding data collection. A retrospective cohort design was selected to assess real-world clinical outcomes associated with both surgical techniques within routine orthopedic practice.

Ethical approval was obtained from the Institutional Ethics Committee prior to commencement of the study (Approval No. PMU/PMCH/IEC/GEN/2026/297; dated 31 March 2026). The study was conducted in accordance with the principles of the Declaration of Helsinki. Given the retrospective nature of the investigation and the use of anonymized patient records, the requirement for informed consent was waived by the Ethics Committee.

Study population

Medical records of consecutive patients who underwent primary TKA for end-stage knee osteoarthritis during the study period were reviewed. A total of 301 consecutive eligible patients fulfilled the predefined inclusion and exclusion criteria and were included in the final analysis. Of these, 87 patients underwent robotic-assisted TKA and 214 underwent conventional TKA. Treatment allocation was not randomized and reflected routine clinical practice based on surgeon recommendations, patient preference, availability of the robotic platform, and clinical suitability during the study period. The unequal group sizes reflect routine clinical practice during the study period, as robotic-assisted TKA was progressively introduced and therefore performed less frequently than conventional TKA. To reduce potential selection bias associated with this imbalance, multivariable regression analysis and propensity score matching (1:2 nearest-neighbor matching) were performed.

Patients were eligible for inclusion if they: (1) had radiographically confirmed primary knee osteoarthritis, (2) underwent unilateral primary TKA, (3) had complete preoperative and postoperative clinical records, and (4) completed at least one year of follow-up. Patients undergoing revision TKA, simultaneous bilateral arthroplasty, arthroplasty for inflammatory arthritis or post-traumatic arthritis, or those with incomplete records were excluded.

Data collection

Demographic, clinical, and perioperative data were extracted from electronic medical records and operative databases. Baseline variables included age, sex, body mass index (BMI), Kellgren-Lawrence (KL) grade, American Society of Anesthesiologists (ASA) classification, diabetes mellitus, hypertension, coronary artery disease, and preoperative functional status.

Preoperative functional assessment included the Knee Society Score (KSS) [9], Western Ontario and McMaster Universities Osteoarthritis Index (WOMAC) [10], Oxford Knee Score (OKS) [11], Forgotten Joint Score (FJS-12) [12], and knee range of motion (ROM) [13,14].

Surgical technique

The same group of experienced arthroplasty surgeons performed both robotic-assisted and conventional procedures using standardized perioperative care pathways, thereby minimizing variability related to surgeon experience and postoperative management. Both robotic-assisted and conventional procedures were routinely performed during the study period using standardized institutional protocols.

In the robotic-assisted group, procedures were performed using the CUVIS-joint active robotic system (Curexo Inc., Seoul, South Korea), marketed in India by Meril Life Sciences Pvt. Ltd., Vapi, Gujarat, India. The system incorporates a robotic arm, preoperative planning software, intraoperative registration, and real-time navigation to facilitate accurate bone resection, implant positioning, and soft-tissue balancing [15,16]. Surgical planning was individualized for each patient, and component placement was executed under robotic guidance.

Patients in the conventional group underwent TKA using standard intramedullary and extramedullary alignment guides according to established institutional protocols. Implant selection and surgical approach were based on the surgeon's preference and patient-specific requirements.

Postoperative rehabilitation protocols, thromboprophylaxis, antibiotic prophylaxis, pain management, and discharge criteria were standardized across both groups.

Outcome measures

Perioperative Outcomes

Perioperative outcomes assessed included operative time, tourniquet time, estimated blood loss, postoperative hemoglobin reduction, and length of hospital stay (LOS).

Functional outcomes

Functional outcomes were assessed using validated patient-reported and clinician-reported outcome measures, including the Knee Society Score (KSS) [9], Western Ontario and McMaster Universities Osteoarthritis Index (WOMAC) [10], Oxford Knee Score (OKS) [11], Forgotten Joint Score-12 (FJS-12) [12], and knee range of motion (ROM) measured using standard goniometric techniques [13].

Assessments were performed preoperatively and during postoperative follow-up at 6 weeks, 3 months, and 12 months. Higher KSS, OKS, FJS-12, and ROM values indicated superior clinical outcomes, whereas lower WOMAC scores reflected better functional status. No modifications were made to the original validated instruments. The outcome measures were used in accordance with their published validation studies and for non-commercial academic research purposes.

Minimal clinically important differences (MCID)

To interpret the clinical relevance of observed differences, established MCID thresholds were applied: KSS: 8-10 points [17]; OKS: 5 points [18]; WOMAC: 10-15 points [19]; FJS-12: 10-12 points [20]; ROM: 5 degrees [21]. Differences exceeding these thresholds were considered clinically meaningful, regardless of statistical significance.

Postoperative complications and patient satisfaction

Overall patient satisfaction was assessed at the 12-month follow-up using routine institutional postoperative records and categorized as satisfied or not satisfied. Postoperative complications were defined as any documented adverse event requiring medical or surgical management during the follow-up period, including surgical site infection, deep vein thrombosis, periprosthetic fracture, readmission, revision surgery, or reoperation.

Data management and quality control

Patient records were reviewed for completeness before inclusion. Cases with missing key demographic, perioperative, or outcome variables were excluded from the analysis (complete-case analysis). No imputation of missing data was performed because only patients with complete datasets fulfilling the predefined eligibility criteria were included. Data accuracy was verified through cross-checking with operative records and clinical documentation. Continuous variables were assessed for plausibility and consistency before statistical analysis.

Statistical analysis

Statistical analyses were performed using IBM SPSS Statistics for Windows, Version 25.0 (IBM Corp., Armonk, NY, USA).

Continuous variables were summarized as mean ± standard deviation (SD), whereas categorical variables were presented as frequencies and percentages. Comparisons between the robotic-assisted and conventional groups were performed using the independent-samples t-test for continuous variables. Categorical variables were compared using the chi-square test or Fisher's exact test, as appropriate.

To identify independent predictors of early postoperative functional recovery, multivariable linear regression analysis was performed using the three-month Knee Society Score as the dependent variable. Variables entered into the model included surgical technique, age, body mass index, Kellgren-Lawrence grade, diabetes mellitus, preoperative Knee Society Score (KSS), and preoperative range of motion. Regression coefficients (β), 95% confidence intervals (CIs), and p-values were reported.

Matching variables included age, sex, BMI, ASA classification, Kellgren-Lawrence grade, diabetes mellitus, hypertension, coronary artery disease, preoperative KSS, WOMAC score, and ROM. To minimize the selection bias inherent to retrospective observational studies, propensity score matching (PSM) was performed using a 1:2 nearest-neighbor matching approach without replacement. Matching variables included age, sex, BMI, ASA classification, Kellgren-Lawrence grade, diabetes mellitus, hypertension, coronary artery disease, preoperative KSS, WOMAC score, and ROM. Post-matching analyses were subsequently conducted to compare perioperative outcomes, functional outcomes, complications, and revision rates between the two groups. A caliper width of 0.2 standard deviations of the logit of the propensity score was applied. Covariate balance was assessed using standardized mean differences (SMDs), with values <0.10 considered indicative of adequate balance. Post-matching analyses were subsequently conducted to compare perioperative outcomes, functional outcomes, complications, and revision rates between the two groups.

Covariate balance before and after matching was assessed using standardized mean differences (SMDs), with values <0.10 indicating adequate balance. Regression assumptions, including linearity, normality of residuals, homoscedasticity, and multicollinearity, were evaluated before fitting the multivariable linear regression model and were found to be satisfactory.

The distribution of continuous variables was assessed using visual inspection of histograms and the Shapiro-Wilk test. Normally distributed variables were compared using the independent-samples t-test, whereas categorical variables were analyzed using the chi-square test or Fisher's exact test, as appropriate.

Post-hoc power analysis

Post-hoc power calculations were performed using two-tailed α = 0.05. For continuous outcomes, the observed power was calculated based on the detected effect sizes. For the 12-month KSS (90±8 vs 89±8; difference 1.0 point), the achieved power was 18.8%, indicating that the study was underpowered to detect a difference of this magnitude. With the available sample sizes (n=87 robotic, n=214 conventional), the study had 80% power to detect a minimum difference of 3.5 points in KSS.

For binary outcomes, achieved power was calculated using observed event rates. For complications (3.45% vs 3.74%), the achieved power was 6.1%. For revision surgery (1.15% vs 1.40%), the achieved power was 5.4%. For patient satisfaction (90.8% vs 88.3%), the achieved power was 19.7%. The study was adequately powered only for large absolute differences (≥7.5% for complications, ≥9.5% for satisfaction).

Due to low event rates, conclusions of equivalence for rare events (complications, revision surgery) are not supported by the present sample size. These findings should be considered hypothesis-generating.

All statistical tests were two-tailed, and a p-value <0.05 was considered statistically significant.

## Results

Study cohort and baseline characteristics

**Figure 1:**
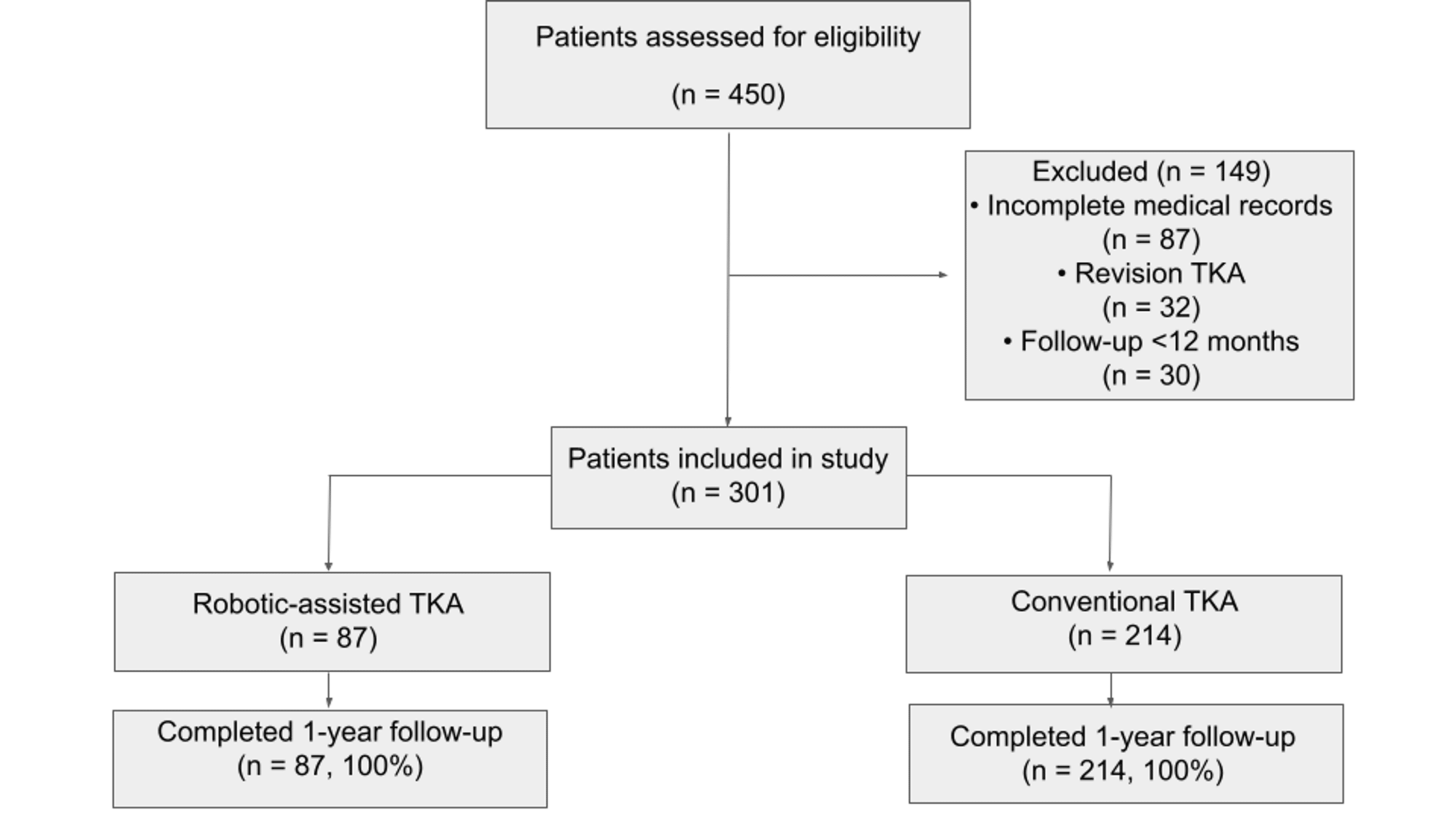
Flowchart illustrating patient screening, exclusions, final cohort allocation, and completion of the minimum one-year follow-up period

A total of 301 consecutive patients who underwent primary TKA and fulfilled the study eligibility criteria were included in the final analysis (Figure 1). Of these, 87 patients underwent robotic-assisted TKA (RA-TKA) and 214 underwent conventional TKA (C-TKA). Baseline demographic and clinical characteristics were comparable between groups (Table 1).

**Table 1:** Baseline demographic and clinical characteristics Values are presented as mean ± standard deviation (SD) or number (N, %). Continuous variables were compared using the independent samples t-test. Categorical variables were compared using the chi-square (χ²) test. Statistical significance was defined as p < 0.05. TKA: total knee arthroplasty; KL: Kellgren-Lawrence; ASA: American Society of Anesthesiologists; KSS: Knee Society Score; WOMAC: Western Ontario and McMaster Universities Osteoarthritis Index; OKS: Oxford Knee Score; FJS: Forgotten Joint Score; ROM: range of motion

Variable	Robotic TKA (N=87)	Conventional TKA (N=214)	Test Statistic	P value
Age (years)	66.2 ± 7.1	67.1 ± 7.8	t=0.95	0.34
Male Sex, N (%)	28 (32.2%)	71 (33.2%)	χ²=0.03	0.87
BMI (kg/m²)	29.8 ± 3.9	30.2 ± 4.2	t=0.81	0.42
KL Grade III, N (%)	25 (28.7%)	58 (27.1%)	χ²=0.07	0.79
KL Grade IV, N (%)	62 (71.3%)	156 (72.9%)	—	—
ASA Grade III, N (%)	22 (25.3%)	55 (25.7%)	χ²=0.01	0.91
Diabetes Mellitus, N (%)	22 (25.3%)	58 (27.1%)	χ²=0.10	0.75
Hypertension, N (%)	43 (49.4%)	111 (51.9%)	χ²=0.16	0.69
Coronary Artery Disease, N (%)	7 (8.0%)	20 (9.3%)	χ²=0.12	0.73
Preoperative KSS	42.8 ± 9.4	43.1 ± 10.2	t=0.24	0.81
Preoperative WOMAC	57.6 ± 11.8	58.2 ± 12.4	t=0.36	0.72
Preoperative OKS	19.8 ± 5.3	20.1 ± 5.6	t=0.40	0.69
Preoperative FJS	12.4 ± 6.2	12.1 ± 6.5	t=0.31	0.76
Preoperative ROM (°)	98 ± 12	97 ± 13	t=0.59	0.55

No statistically significant differences were observed with respect to age, sex distribution, body mass index, Kellgren-Lawrence grade, ASA classification, or comorbidity profile. Similarly, preoperative functional status, including KSS, WOMAC, OKS, FJS-12, and ROM, did not differ significantly between the two cohorts, indicating a balanced baseline population for subsequent comparisons.

Perioperative outcomes

Perioperative outcomes are summarized in Table 2. Robotic-assisted procedures required significantly longer operative and tourniquet times than conventional TKA. However, RA-TKA was associated with lower intraoperative blood loss, a smaller postoperative decrease in hemoglobin, and a shorter length of hospital stay. These findings indicate improved perioperative recovery despite the longer surgical duration associated with robotic assistance.

**Table 2:** Operative and Perioperative Outcomes Values are presented as mean ± SD. An independent samples t-test was used. Statistical significance was defined as p < 0.05. TKA: total knee arthroplasty

Variable	Robotic TKA (N=87)	Conventional TKA (N=214)	Test Statistic	P value
Operative Time (minutes)	108.4 ± 14.2	88.6 ± 11.5	t=11.2	<0.001
Tourniquet Time (minutes)	78.2 ± 10.1	72.4 ± 9.8	t=3.12	0.002
Blood Loss (mL)	255 ± 92	289 ± 105	t=2.18	0.03
Hemoglobin Drop (g/dL)	1.9 ± 0.7	2.2 ± 0.8	t=2.57	0.01
Length of Stay (days)	4.2 ± 1.1	4.8 ± 1.3	t=2.61	0.01

Functional outcomes

**Figure 2:**
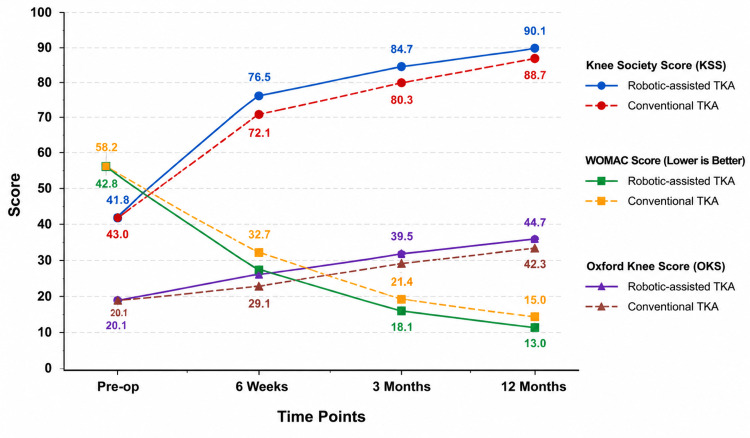
Functional outcome trends following total knee arthroplasty Mean Knee Society Score (KSS), Western Ontario and McMaster Universities Osteoarthritis Index (WOMAC), and Oxford Knee Score (OKS) values are displayed at baseline, 6 weeks, 3 months, and 12 months postoperatively. Error bars represent standard deviations. Higher KSS and OKS values indicate superior outcomes, whereas lower WOMAC values indicate superior outcomes. Statistical significance was defined as p < 0.05.

Both groups demonstrated substantial postoperative improvement across all functional outcome measures during follow-up (Table 3, Figure 2). At six weeks and three months, patients undergoing RA-TKA achieved significantly better functional outcomes than those undergoing conventional TKA, as reflected by higher KSS, OKS, and FJS-12 scores and lower WOMAC scores.

**Table 3:** Patient-reported outcome measures Values are presented as mean ± SD. An independent samples t-test was used for between-group comparisons. Statistical significance was defined as p < 0.05. TKA: total knee arthroplasty; KL: Kellgren-Lawrence; ASA: American Society of Anesthesiologists; KSS: Knee Society Score; WOMAC: Western Ontario and McMaster Universities Osteoarthritis Index; OKS: Oxford Knee Score; FJS: Forgotten Joint Score; ROM: range of motion

Outcome Measure	Robotic TKA	Conventional TKA	Test Statistic	P value
KSS (6 weeks)	76.5 ± 8.2	72.1 ± 8.8	t=2.91	0.004
KSS (3 months)	84.7 ± 7.4	80.3 ± 8.2	t=3.08	0.002
KSS (12 months)	90 ± 8	89 ± 8	t=1.08	0.28
WOMAC (6 weeks)	28.3 ± 9.4	32.7 ± 10.2	t=2.59	0.01
WOMAC (3 months)	18.1 ± 7.8	21.4 ± 8.5	t=2.33	0.02
WOMAC (12 months)	13 ± 7	15 ± 8	t=1.56	0.12
OKS (6 weeks)	31.8 ± 6.5	29.1 ± 6.8	t=2.18	0.03
OKS (3 months)	39.5 ± 5.4	36.9 ± 5.8	t=2.59	0.01
FJS (6 weeks)	39.2 ± 12.4	34.1 ± 11.8	t=2.34	0.02
FJS (3 months)	58.4 ± 15.1	51.2 ± 14.6	t=2.57	0.01
ROM at 12 months (°)	124 ± 8	122 ± 9	t=1.44	0.15

The greatest between-group differences were observed during the early postoperative period, suggesting enhanced short-term functional recovery in the robotic-assisted cohort. By 12 months, functional outcomes had improved markedly in both groups, and differences in KSS, WOMAC, and ROM were no longer statistically significant, indicating convergence of long-term clinical outcomes.

Complications and patient satisfaction

Postoperative complications were infrequent in both groups (Table 4). Rates of surgical site infection, deep vein thrombosis, periprosthetic fracture, readmission, and reoperation were low. No statistically significant differences were detected between groups; however, post-hoc power analysis indicated that the study was underpowered for these rare events (achieved power 5-6%). Therefore, these findings are hypothesis-generating and require confirmation in larger cohorts.

**Table 4:** Complications and patient satisfaction Values are presented as numbers (N, %). Chi-square (χ²) or Fisher's Exact test was used as appropriate. Statistical significance was defined as p < 0.05.

Variable	Robotic TKA N (%)	Conventional TKA N (%)	Test Statistic	P value
Patient Satisfaction	79 (91.0%)	189 (88.3%)	χ²=1.16	0.31
Surgical Site Infection	1 (1.1%)	4 (1.9%)	Fisher	0.65
Deep Vein Thrombosis	1 (1.1%)	3 (1.4%)	Fisher	0.83
Periprosthetic Fracture	0 (0.0%)	1 (0.5%)	Fisher	0.52
Revision Surgery	1 (1.1%)	3 (1.4%)	Fisher	0.87
Readmission	2 (2.3%)	7 (3.3%)	χ²=0.17	0.68
Reoperation	1 (1.1%)	4 (1.9%)	Fisher	0.65

Multivariable analysis of early functional recovery

Results of the multivariable linear regression analysis are presented in Table 5. After adjustment for demographic and clinical covariates, robotic-assisted TKA remained independently associated with improved three-month KSS. Higher preoperative KSS and greater preoperative ROM were also significant positive predictors of postoperative functional recovery. Conversely, increased BMI was associated with lower three-month KSS. Age, diabetes mellitus, and advanced radiographic disease severity were not independently associated with postoperative functional outcomes.

**Table 5:** Multivariable linear regression analysis for the three-month Knee Society Score Multiple linear regression model evaluating independent predictors of three-month KSS. β coefficients represent adjusted effect estimates. Statistical significance was defined as p < 0.05. KL: Kellgren-Lawrence; KSS: Knee Society Score; ROM: range of motion

Variable	β Coefficient	95% Confidence Interval	t Statistic	P value
Robotic-Assisted TKA	+3.82	1.24 to 6.40	2.95	0.004
Age (years)	-0.11	-0.24 to 0.02	-1.67	0.09
BMI (kg/m²)	-0.19	-0.34 to -0.04	-2.52	0.01
KL Grade IV	-0.58	-1.92 to 0.76	-0.84	0.40
Diabetes Mellitus	-0.72	-2.11 to 0.67	-1.01	0.31
Preoperative KSS	+0.44	0.33 to 0.55	7.92	<0.001
Preoperative ROM	+0.15	0.04 to 0.26	2.71	0.007

Propensity score-matched analysis

To account for the potential baseline selection bias, a 1:2 propensity score-matched analysis was performed (Table 6). Following matching, robotic-assisted TKA continued to demonstrate superior early functional outcomes, including improved KSS, WOMAC, OKS, and FJS-12 scores at three months. Length of hospital stay also remained significantly shorter in the robotic-assisted cohort.

**Table 6:** Propensity Score-Matched Outcome Analysis (1:2 Matching) One-to-two nearest-neighbor propensity score matching was performed using age, sex, BMI, ASA class, KL grade, diabetes mellitus, hypertension, coronary artery disease, preoperative KSS, WOMAC, and ROM. Values are presented as mean ± SD or N (%). Statistical significance was defined as p < 0.05. TKA: total knee arthroplasty; KL: Kellgren-Lawrence; ASA: American Society of Anesthesiologists; KSS: Knee Society Score; WOMAC: Western Ontario and McMaster Universities Osteoarthritis Index; OKS: Oxford Knee Score; FJS: Forgotten Joint Score; ROM: range of motion

Outcome	Robotic TKA (N=87)	Conventional TKA (N=174)	Test Statistic	P value
Length of Stay (days)	4.2 ± 1.1	4.6 ± 1.2	t=2.31	0.02
KSS (3 months)	84.7 ± 7.4	80.9 ± 7.9	t=2.87	0.005
WOMAC (3 months)	18.1 ± 7.8	21.0 ± 8.2	t=2.22	0.03
OKS (3 months)	39.5 ± 5.4	37.2 ± 5.7	t=2.46	0.01
FJS (3 months)	58.4 ± 15.1	52.3 ± 14.4	t=2.39	0.02
Complications	3 (3.4%)	8 (4.6%)	χ²=0.18	0.67
Revision Surgery	1 (1.1%)	2 (1.1%)	Fisher	0.99

No significant differences were observed in complication rates or revision surgery following matching. The persistence of these findings after adjustment for baseline characteristics supports the robustness of the observed association between robotic assistance and enhanced early postoperative recovery.

## Discussion

The present retrospective cohort study compared the clinical outcomes of RA-TKA and C-TKA in patients undergoing primary TKA for end-stage knee osteoarthritis. The principal findings of this study were that RA-TKA was associated with superior early postoperative functional recovery, reduced intraoperative blood loss, lower postoperative hemoglobin reduction, and shorter hospital stay, albeit at the expense of longer operative and tourniquet times. However, long-term functional outcomes, complication rates, revision rates, and patient satisfaction were largely comparable between the two groups. These findings suggest that the primary clinical benefit of robotic assistance may be reflected in enhanced early recovery rather than substantial long-term superiority.

The unequal sample sizes between treatment groups were expected in this retrospective real-world cohort because robotic-assisted TKA represented a newer technology that was introduced gradually into routine practice. To account for this imbalance, we employed multivariable adjustment and propensity score matching, both of which yielded results consistent with the primary analyses.

Both RA-TKA and C-TKA resulted in significant postoperative improvements across all functional outcome measures, confirming the effectiveness of TKA in restoring function and alleviating disability associated with advanced knee osteoarthritis. Nevertheless, patients in the robotic-assisted cohort demonstrated significantly better KSS, WOMAC, OKS, and FJS-12 scores during the early postoperative period. However, it is important to note that none of these observed differences exceeded established MCID thresholds: KSS difference 4.4 points (MCID: 8-10 points [17]), OKS difference 2.6 points (MCID: 5 points [18]), WOMAC difference 3.3 points (MCID: 10-15 points [19]), and FJS-12 difference 7.2 points (MCID: 10-12 points [20]). These findings are consistent with contemporary systematic reviews and meta-analyses that have reported improved early functional recovery following robotic-assisted procedures, potentially attributable to more accurate component positioning, individualized alignment strategies, and improved soft-tissue balancing [2,4-6,16]. Robotic systems facilitate real-time intraoperative assessment and execution of surgical plans with a high degree of precision, reducing technical variability and improving reproducibility of implant placement [1,5,14]. Such advantages may contribute to enhanced early joint function and patient-perceived recovery following surgery.

An important observation in the present study was the significantly longer operative time associated with robotic-assisted procedures. This finding is consistent with previous reports and reflects the additional steps required for image acquisition, registration, intraoperative planning, and robotic system setup [1,4,5]. The learning curve associated with robotic technology may further contribute to increased operative duration, particularly during the early adoption phase. Despite this limitation, the robotic cohort experienced lower blood loss, smaller postoperative hemoglobin declines, and shorter hospitalization. These findings support the hypothesis that robotic assistance may reduce surgical trauma through more controlled bone preparation and improved soft-tissue preservation [1,15]. Earlier mobilization and accelerated postoperative recovery may therefore offset some of the disadvantages associated with prolonged operative times.

The present study also demonstrated that complication rates were low and comparable between groups. No significant differences were observed with respect to infection, thromboembolic events, revision surgery, readmission, or reoperation. These findings are generally consistent with recent systematic reviews reporting low complication rates following robotic-assisted TKA; however, the present study was underpowered to detect small differences in rare adverse events [2,4-6,16]. Similarly, patient satisfaction rates were high in both groups and did not differ significantly. This observation is particularly relevant because patient satisfaction remains one of the most important indicators of success following TKA, with previous studies reporting dissatisfaction rates approaching 20% despite technically successful procedures [3].

Interestingly, although robotic-assisted TKA demonstrated superior short-term functional outcomes, the magnitude of these differences diminished during longer follow-up. By one year, outcomes between the two groups had largely converged. Similar findings have been reported in several contemporary meta-analyses, which consistently demonstrate improved radiographic accuracy and alignment precision with robotic assistance but fail to identify substantial long-term differences in patient-reported outcomes or implant survival [2,5,6,16]. The discrepancy between improved surgical precision and equivalent long-term outcomes may reflect the multifactorial nature of recovery after TKA. Factors such as patient expectations, rehabilitation adherence, muscle strength, comorbidities, and psychosocial influences may ultimately exert a greater impact on long-term outcomes than minor differences in component positioning alone.

Multivariable regression analysis provided additional insight into determinants of postoperative recovery. Robotic-assisted surgery remained an independent predictor of improved three-month KSS after adjustment for potential confounders, suggesting that the observed benefits were not solely attributable to baseline differences between groups. Furthermore, higher preoperative KSS and greater preoperative ROM were associated with better postoperative outcomes, whereas increased BMI adversely influenced recovery. These findings reinforce the importance of preoperative optimization and patient selection in achieving favorable arthroplasty outcomes.

To further address potential selection bias, propensity score matching was performed. The persistence of superior early functional outcomes in the matched cohort strengthens the validity of the findings and suggests that robotic assistance contributed independently to improved postoperative recovery. Similar observations have been reported in real-world clinical studies demonstrating improved early outcomes and recovery trajectories following robotic-assisted TKA while maintaining comparable safety profiles [7].

From a clinical perspective, the findings of this study suggest that robotic-assisted TKA was associated with modest improvements in early postoperative recovery and selected perioperative outcomes. However, these early functional differences did not exceed established MCID thresholds and should therefore be interpreted cautiously. Furthermore, owing to the retrospective observational design, these findings demonstrate an association rather than a causal effect of robotic assistance. These advantages may be valuable in contemporary enhanced recovery programs that emphasize early mobilization, shorter hospitalization, and improved patient experience. However, the absence of clear long-term superiority highlights the importance of considering economic factors, resource utilization, and institutional infrastructure when evaluating the adoption of robotic systems [1].

Future multicenter prospective studies with longer follow-up durations are needed to determine whether the early clinical advantages observed with robotic-assisted TKA translate into superior implant longevity, improved cost-effectiveness, and sustained patient satisfaction. Further research should also explore patient subgroups most likely to benefit from robotic technology and investigate the relationship between surgical precision, alignment strategies, and long-term functional outcomes.

Limitations

Several limitations should be acknowledged. First, the retrospective single-center design may introduce residual confounding despite multivariable adjustment and propensity score matching. Second, treatment allocation was not randomized, and the unequal sample sizes between the robotic-assisted and conventional TKA groups reflect routine clinical practice rather than planned allocation, potentially introducing selection bias. However, propensity score matching and adjusted regression analyses were performed to minimize this bias. In addition, functional outcomes at different follow-up time points were analyzed using between-group comparisons rather than repeated-measures modeling. Consequently, within-patient longitudinal changes over time may not have been fully captured. Third, the follow-up period may be insufficient to evaluate long-term implant survivorship and late complications. Fourth, post-hoc power analysis revealed that the study was underpowered to detect small differences in complication rates and revision surgery. The observed revision rate of 1.1-1.4% would require approximately 1,500 patients per group to achieve 80% power. Consequently, the absence of statistically significant differences in these rare outcomes should not be interpreted as evidence of safety equivalence, and larger multicenter studies are needed. Fifth, the unequal sample sizes between the robotic-assisted and conventional TKA groups reflected routine clinical practice and may have introduced imbalance despite statistical adjustment. Sixth, although all procedures were performed by experienced arthroplasty surgeons using standardized institutional protocols, differences related to the surgeon's learning curve and center-specific expertise with robotic technology may have influenced perioperative and functional outcomes. Seventh, outcome assessment was not blinded because of the retrospective study design, introducing the possibility of measurement bias for subjective outcomes such as functional scores and patient satisfaction. Eighth, although multivariable regression and propensity score matching were performed to reduce selection bias and measured confounding, residual confounding from unmeasured variables cannot be completely excluded. Ninth, radiographic alignment parameters and component positioning were not evaluated, limiting assessment of the relationship between surgical accuracy and clinical outcomes. Finally, cost-effectiveness analyses were beyond the scope of the present investigation and warrant further study. Although multivariable adjustment and propensity score matching improved the comparability of the study groups, the possibility of residual confounding from unmeasured variables remains an inherent limitation of retrospective observational studies and should be considered when interpreting the findings.

## Conclusions

Robotic-assisted total knee arthroplasty was associated with modest improvements in early postoperative recovery compared with conventional total knee arthroplasty, including better short-term functional outcomes, reduced blood loss, lower postoperative hemoglobin reduction, and shorter hospital stay. Although these early differences were statistically significant, they did not exceed established minimal clinically important differences thresholds and should be interpreted within the context of the retrospective observational study design. Although robotic procedures were associated with longer operative times, both techniques achieved substantial clinical improvement. No statistically significant differences were observed in complications, revision surgery, patient satisfaction, or one-year functional outcomes; however, the study was underpowered to detect small differences in rare adverse events. These findings suggest that robotic-assisted total knee arthroplasty may offer modest advantages in early postoperative recovery; however, long-term functional outcomes, complication rates, revision surgery, and patient satisfaction were comparable within the limitations of the present study.

This study contributes real-world evidence from a high-volume orthopedic center and demonstrates that the early advantages of robotic-assisted total knee arthroplasty remain evident even after adjustment for potential confounding factors through propensity score matching. As robotic technology continues to evolve, further prospective multicenter studies with longer follow-up and cost-effectiveness analyses are required to determine whether the modest early recovery advantages observed in this study translate into clinically meaningful long-term benefits and broader healthcare value.
